# Muscle Thickness and Echo Intensity by Ultrasonography and Cognitive and Physical Dimensions in Older Adults

**DOI:** 10.3390/diagnostics11081471

**Published:** 2021-08-13

**Authors:** Alvaro Mateos-Angulo, Alejandro Galán-Mercant, Antonio Ignacio Cuesta-Vargas

**Affiliations:** 1 Department of Physiotherapy, IBIMA, University of Málaga, 29017 Malaga, Spain; amateos@uma.es (A.M.-A.); acuesta@uma.es (A.I.C.-V.); 2 MOVE-IT Research Group and Department of Physical Education, Department of Nursing and Physiotherapy, Cádiz University, 11009 Cádiz, Spain; 3 Biomedical Research and Innovation Institute of Cádiz (INiBICA) Research Unit, Puerta del Mar University Hospital, University of Cádiz, 11009 Cádiz, Spain; 4 School of Clinical Sciences of the Faculty of Health, Queensland University of Technology, Brisbane 4000, Australia

**Keywords:** sarcopenia, muscle ultrasound, muscle quality, physical cognitive performance

## Abstract

The purpose of the present study was to investigate the associations between muscle thickness and echo intensity with cognitive and physical dimensions like functional capacity measured in older people. This cross-sectional study involved 20 older adults (15 women and 5 men, mean age ± SD: 85 ± 7 years, body mass index: 25 ± 3 kg/m^2^) from a geriatric centre in Malaga (Spain). Anthropometric measurements, cognitive assessment with Pfeiffer Short Portable Mental Status Questionnaire and Motor Memory test, Physical Performance with Short Physical Performance Battery, and muscle strength were tested. Additionally, using B-mode ultrasonography, images of wrist flexors, biceps brachii, rectus femoris, vastus lateralis, medial gastrocnemius, and tibialis anterior were captured, and muscle thickness and echo intensity variables were extracted. An association between muscle parameters assessed by ultrasonography and cognitive and physical dimensions were found in older people. Echo intensity was the best predictor in a set of regression models with different muscle parameters and a battery of cognitive and physical tests in older people. Echo intensity adjusted by handgrip strength could be a low cost and ambulatory index and an indirect and reversible indicator of functional capacity.

## 1. Introduction

Ageing is a process associated with multiple changes, including the neuromuscular system, which is fundamental to preserve functional capacity. Sarcopenia was defined as the loss of muscle mass resulting from age [1,2]. Sarcopenia directly affects an individual’s health status and is related to mobility problems, loss of independence and less ability to carry out daily living activities, and a higher risk of falls and increased comorbidity [3,4,5]. Recently, the diagnosis criteria of sarcopenia have been revised, including three parameters: low muscle strength, low muscle quantity or quality, and low physical performance in severe cases [6]. Muscle quality has been defined as muscle strength or power per unit of muscle mass [7] and is one of the strongest predictors of functional capacity in older adults [8]. Muscle quality is commonly affected with aging by the accumulation of non-contractile tissues in muscle mass [3,5], and this condition can be evaluated by dual-energy X-ray absorptiometry (DXA), computed tomography (CT), or ultrasonography [8].

Brightness mode (B-mode) muscle ultrasound is a safe and low-cost technique for the measurement of muscle architecture across the muscular thickness (MT) and echo intensity (EI) [9]. However, the evaluation of age-related changes in muscle mass by MT assessment could result in an overestimation of the amount of contractile muscle in older adults [10]. Muscle quality can be evaluated by EI on ultrasonography images of skeletal muscle in combination with computer-aided grey-scale analysis [11,12]. The deposition of adipose and connective tissue with aging can affect muscle quality, and this condition is reflected on ultrasound images with increasing EI values and results in a decrease in muscle function [13]. A relationship in older adults between EI and physical function variables like strength [14], gait speed [15] or sit-to-stand ability [15,16] have been shown. Previous studies suggest physiological relations between cognitive and physical function [16]. Physical function decline could be a marker of neural system deterioration [17], and physical function level and cognitive status are related with disease factors [16]. For these reasons, it could be interesting to explore relations between muscle EI, physical function, and cognitive status.

Previous studies have investigated the relationship between EI and muscular strength [10,18,19] and functional tests [15,20,21,22,23,24]. However, relationships between muscle ultrasound parameters and cognitive and physical dimensions across cognitive and physical functions tests have not been investigated in this context. More evidence is necessary to explore clinical implications of muscle ultrasound parameters in geriatric assessment. The aim of the present study was to investigate the relationships and level of correlation between MT and EI with cognitive and physical dimensions like functional capacity measured in institutionalized older adults.

## 2. Methods

### 2.1. Study Participants

A total of 20 older adults (15 women and 5 men, mean age ± SD: 85 ± 7 years, body mass index: 25 ± 3 kg/m^2^) were recruited between February and June 2018 from a geriatric centre. Inclusion criteria were: males and females who were institutionalized older adults, aged between 70–100 years. Exclusion criteria were: neurological dysfunction, neuromuscular pathology, and any condition that prevented the completion of tests or any pathology that could be aggravated by participating in the study. Each participant received a detailed explanation of the study and gave written informed consent before participation. The study complied with the principles laid out in the Declaration of Helsinki and was approved by the ethics committee of the Faculty of Health Sciences at the local University.

### 2.2. Procedures

Study participants were evaluated in two visit days. On the first day, participants performed cognitive and physical tests. On the second day, we collected in the following order: anthropometrics, ultrasound assessment, and muscle strength variables. The time lapse between the two measurements visit days was at least two days apart and less than one week.

### 2.3. Anthropometric Measurements

Height, weight, calf circumference, and mid-upper arm circumference were recorded following the guidelines of The International Society for the Advancement of Kinanthropometry (ISAK) [25].

### 2.4. Cognitive Assessment

To evaluate the cognitive status of the participants, the Spanish version of Pfeiffer SPMSQ [26] was used. The SPMSQ consist of 10 questions; if 0–2 questions were wrong, the participants were classified as normal mental functioning; 3–4 errors at mild cognitive impairment; 5–7 errors at moderate cognitive impairment; and 8 or more errors at severe cognitive impairment. The Spanish version of this questionnaire was validated previously [27].

Motor memory and body awareness were evaluated through a test consisting of the reproduction of ten static postures. The examiner performs 10 postures, keeping each one for 10 s. After that time, the patient reproduces the posture in a contralateral way, as if it were a mirror. The test ends when the 10 postures have been made. This test is part of the “Memory in Motion” program, used as a cognitive intervention tool [28,29].

### 2.5. Physical Performance

The SPPB test was used to assess functional capacity [30]. The SPPB test includes three objective physical tests: balance tandem test, 4 metres gait speed, and sit-to-stand test. The balance test consists of maintaining balance in side-by-side, semi-tandem (heel of one foot beside the big toe of the other foot), and tandem (heel of one foot directly in front of the other foot) positions. The gait speed was recorded as the time used to walk 4 metres (the best of two attempts was recorded). In the sit-to-stand test, participants in a sitting position in a chair and folding their arms across their chests were asked to stand up and sit down five times as quickly as possible. Every test scored from 0 to 4. SPPB total score (0–12) was obtained by adding the scores of each test. Higher scores involve better functional capacity [30]. Gait speed in metres per second (m/s) was also recorded as independent variable for analysis.

In addition, the TUG test was performed by participants. The TUG is a test used to evaluate functional mobility in older adults [31]. The time used to stand up from a chair, walk three metres, and come back to sit down in the chair was recorded (the best time of two attempts was chosen). Tinetti Performance-Oriented Mobility Assessment test [32] and Barthel Index [33] were also evaluated.

### 2.6. Muscle Strength

Bilateral handgrip and bilateral knee extensor strength were measured. Handgrip strength (HGS) was calculated using a hydraulic hand-held dynamometer (Saehan Corporation, Masan Free Trade Zone, Korea), which was validated previously [34]. The HGS measurement was assessed following the recommendations of the American Society of Hand Therapists [35]. All participants performed three trials, with one-minute resting, alternating between their dominant and non-dominant hand. The highest value from each side was recorded as maximal grip strength. 

A digital manual dynamometer POWERTRACK^®^ (JtechMedical, Midvale, UT, USA) was used to evaluate knee extensor maximum voluntary contraction (MVC) muscle strength. An interclass correlation coefficient (ICC) ranging from 0.72 to 0.85 was demonstrated for this dynamometer [36]. Knee extensor strength was measured following a protocol previously published [37]. The participant was placed in a sitting position on a stretcher with his hands resting on his legs and feet hanging off the ground. The examiner places one hand to stabilize the subject’s leg and the other hand to support the load cell on the subject’s distal third tibia. Starting from 90° knee flexion, the subject performs a knee extension resisted by the examiner with the dynamometer’s load cell. A full extension must be avoided, with the knee flexion reaching 5°. The maximum peak force was recorded from the dynamometer’s digital display in newtons. The test was performed three times for each participant, with a 2-min break between tests, and the highest value was taken as the knee extensor muscle strength variable.

### 2.7. Muscle Architecture; Muscle Thickness and Echo Intensity

Two longitudinal, static B-mode images on the dominant side of the patient were acquired using Esaote MyLab One (Esaote, Genova, Italy) ultrasound device with a linear array transducer (SL3323) with a variable frequency up to 22 MHz. The following equipment settings were kept at the same level during all examinations: gain (50% of the range) and time gain compensation. Depth was modified during examinations (from 30 to 60 mm) to visualize the entire muscle thickness. A large amount of ultrasound gel was used in order to minimize the pressure applied on the skin and to increase the quality of the images. The same operator carried out a single measurement session to acquire the images.

**Figure 1:**
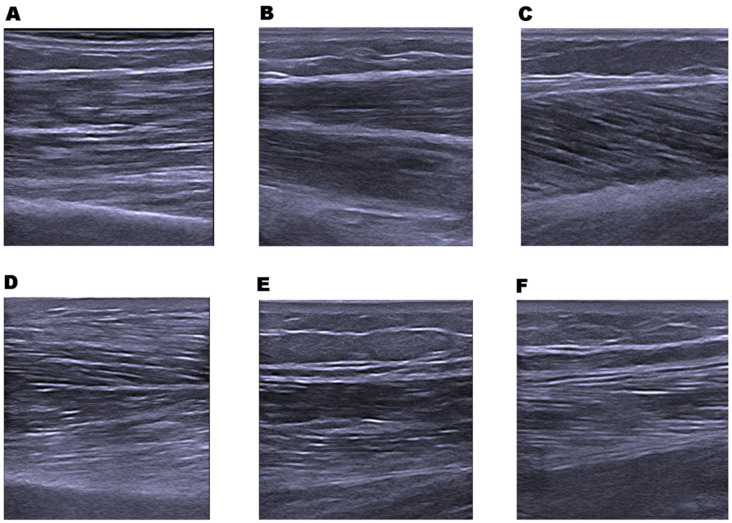
Example of muscle ultrasound images. (**A**) Biceps brachii; (**B**) wrist flexors; (**C**) medial gastrocnemius; (**D**) tibialis anterior; (**E**) rectus femoris; and (**F**) rectus lateralis.

The following upper and lower limb muscles (see Figure 1) of the dominant side were examined using ultrasound B-mode images: superficial wrist flexors muscles (WF), biceps brachii (BB), rectus femoris (RF), vastus lateralis (VL), medial gastrocnemius (MG), and tibialis anterior (TA). These muscles were examined with the participant in the supine position, with the ultrasound operator standing on the ipsilateral side of the participant. MG was examined with the participant in the prone position.

Representation of superficial and deep aponeuroses was optimized in order to achieve the best muscle image possible. The transducer was placed at the anatomical location corresponding to the largest diameter of the muscles examined described in a previous study [11,12]: BB was measured at two-thirds of the distance from the acromion to the antecubital crease; WF at two-fifths of the distance from the antecubital crease to the distal end of the radius; RF half-way along the line from the anterior-superior iliac spine to the superior border of the patella; VL half-way along the line from the anterior-superior iliac spine to the superolateral border of the patella; MG from the mid-sagittal line of the muscle, midway between the proximal and distal tendon insertions and TA at one-quarter of the distance from the inferior aspect of the patella to the lateral malleolus.

Muscle images were analysed offline with ImageJ (NIH Image) software. To calculate the MT, a known distance of 1 cm, as shown in the image, was used to calibrate the software program. The two images were measured and averaged. Muscle thickness was calculated as the distance between the superficial and deep aponeuroses. WF muscle thickness was calculated as the distance between the interosseous membrane (next to the radius) and the superficial fascia of the most ventral flexors of the superficial forearm flexor compartment group. A region of interest was selected including as much area of the muscle as possible but excluding the muscle fascia, using polygon selections for the EI calculation. The EI value was determined by grey-scale analysis function and expressed in arbitrary units as a value between 0 (black) and 255 (white). In addition, mean EI and MT in limbs were calculated using the following equations: MT Upper Limb = [(MT in BB) + (MT in WF)]/2; MT Lower Limb = [(MT in RF) + (MT in RL) + (MT in TA) + (MT in MG)]/4; EI Upper Limb = [(EI in BB) + (EI in WF)]/2; EI Lower Limb = [(EI in RF) + (EI in RL) + (EI in TA) + (EI in MG)]/4.

### 2.8. Statistical Analysis

A database was created with the information gathered from the experimental session in order to analyse the results. The Shapiro–Wilk test was used to explore if the variables were normally distributed. Descriptive statistics were performed with measures of central tendency and dispersion of variables. Inferential statics were performed using Pearson correlation coefficient to determine the association between variables. Linear regression analysis was used to explore the level of contribution of muscle ultrasound variables in the different geriatric tests evaluated. All statistical analyses were performed with SPSS 21 (SPSS Inc., Chicago, IL, USA) software package.

### 2.9. Sample Size

A priori power analysis was conducted with G*Power 3.1, using an effect size of 0.56 observed in a previous study [16] that found relations between ultrasound parameters and functional capacity. The analysis specified a minimum of 18 participants required to achieve a power of 0.80 with α = 0.05.

## 3. Results

Anthropometric, cognitive, physical function, and muscle architecture parameters data are shown in Table 1.

Table 2 shows Pearson coefficient correlations between EI, MT, Barthel test, TUG, GS, SPPB, and muscle strength measurements. Upper limb EI showed a significant negative correlation with knee extensor strength. Lower limb EI showed a significant negative correlation with GS, SPPB, and knee extensor strength. Upper limb MT showed a significant negative correlation with TUG and a significant positive correlation with HGS. Lower limb MT showed a significant positive correlation with Barthel test, SPPB test, STS, HGS, and knee extensor strength.

Table 3 shows a summary of the best stepwise multiple regression analysis carried out in order to identify which variables better predicts functional capacity (SPPB, TUG, gait speed) and cognitive function (motor memory, SPMSQ). Rectus femoris EI adjusted by HGS explains approximately 45% of the variance of SPPB. Medial gastrocnemius EI explains about 30% of the variance of TUG test. Mean EI in LL adjusted by HGS and BMI explain about 73% of GS. Tibialis anterior MT explains about 36% of the variance of motor memory test, and rectus femoris EI explains about 17% of the variance of SPMSQ test. 

## 4. Discussion

The aim of this study was to investigate the association between muscle size (evaluated by ultrasound MT) and muscle quality (evaluated by ultrasound EI) with an integral assessment approach of older adults, including cognitive and physical dimensions like functional capacity. Our results showed significant correlations between muscle parameters assessing by ultrasonography (MT and EI) and physical tests. Our main finding was that EI was the variable most correlated with physical function. We found a regression model in which EI and handgrip strength explains approximately 45% of the variability of the functional capacity in older people, as measured by the SPPB test (*p* < 0.005). On the other hand, our results showed that EI independently explains approximately 30% of the variance of SPPB test (*p* < 0.01). In addition, we found that mean EI in lower limbs explains about 73% of the variance of gait speed adjusted by HGS and BMI, and MG EI explains about 27% of TUG variability. As secondary findings, we found poor correlations between ultrasonography variables and cognitive tests. We found a regression model in which EI explains approximately 17% of the variability of the cognitive domain in older people, as measured by the SPMSQ (*p* < 0.005).

Previous studies reported an association between EI and muscle power [14,15,22,23] and with functional tasks, such as sit-to-stand ability [15,21,22] or gait ability [15,23]. However, this is the first study to investigate the relationship between EI and the SPPB test, which includes a balance test in addition to gait speed and sit-to-stand tests. The SPPB test is popular in clinical practice for evaluating physical performance and functional capacity in older adults, and it has a high health-prognostic value [30,38]. 

Previous studies have found correlations between lower limb MT and knee extensor strength [14,22,24]. The significant correlations between thigh MT and knee extensor strength recorded by Fukumoto (r = 0.47, *p* < 0.01), Wilhelm (r = 0.620, *p* < 0.05), and Strasser (r = 0.834, *p* < 0.01) are similar to the correlations shown in our study between lower limb MT and knee extensor strength (r = 0.495, *p* < 0.05) 

The negative correlations shown in the present study between EI and muscle strength (r = −0.681, *p* < 0.01) were similar to the previous data published by several authors (−0.314 to −0.64; *p* < 0.05) [14,15,19,20,22]. However, Strasser et al. (2013) showed no significant correlations between EI and muscle strength in older people [24].

Rech et al. (2014) found negative correlation between quadriceps EI and gait speed (r = −0.409, *p* < 0.01) [15]. In the same way, the results of the present study showed negative correlations between mean lower limb EI and gait speed (r = −0.556, *p* < = 0.05). In addition, multiple regression analysis showed that EI predicts independently 25% of the variability of gait speed and about 73% after adjusting by HGS and BMI. Osawa et al. (2017) showed a poor relationship between EI of anterior thigh muscles and TUG (β = 0.17, *p* = 0.047) [23]. However, our results showed that MG EI had a relationship with TUG (β = 0.567, *p* < 0.05) but not thigh muscles. The greater relationship showed in our study between MG EI and TUG test could suggest that MG EI could explain better functional mobility than thigh muscles EI. 

The results of the present study showed that EI is correlated with functional capacity better than MT in older people, as measured by the SPPB test, the TUG test, and gait speed test. In the same way, previous studies showed relationship between EI and physical function but not with MT [15,23]. Rech et al. (2014) did not show correlations between MT and functionality when measured by the sit-to-stand test. In addition, Osawa et al. (2016) did not observe a significant association between MT and functional mobility [23]. Better associations between EI and functional capacity than MT suggest that EI can be a more important variable to predict functional loss in older adults than muscle thickness.

The present study is the first to investigate the relationship between EI and the SPPB test. Lopez et al. (2017) investigated quadriceps EI as a predictor of physical performance measured by a 30-s sit-to-stand (30SS) test. A regression model was used to show that EI explained approximately 30% of the variance of the 30SS test [21]. Consistent with Lopez et al., the regression analysis of the present study showed that EI explained independently 30% of the variability of functionality in the older adults. The results of Lopez et al. were limited to the prediction of the 30SS test of functional performance. The SPPB test used in the present study assessed various functional tasks (static and dynamic balance, mobility and gait performance, and functional lower limb power). Hence, these results could have great implications for clinical practice. In addition, another significant multiple regression model involving EI adjusted by handgrip strength explained approximately 45% of the functionality in older adults measured by the SPPB test. This is a high percentage for a functional test that evaluates different physical abilities.

The results of the present study showed that muscle size and muscle quality evaluated by ultrasonography are associated to functional capacity and cognitive status. Aiming to prevent aging decline, the use of ultrasound muscle evaluation in geriatric assessment could be implemented. Muscle quality assessment using echo intensity measurements in addition to handgrip strength evaluation could help to identify people in risk of functional deterioration. Moreover, the associations between echo intensity with physical and cognitive performance could be relevant for the novel concept of motoric cognitive risk syndrome. The motoric cognitive risk syndrome is a recently described clinical situation that relates physical performance and cognitive decline risk [39]. The results of the present study showed that muscle echo intensity is both related to physical and cognitive performance, so muscle echo intensity could be an interesting variable for the assessment of motoric cognitive risk syndrome.

The main limitation of our study was the low sample size, but the distribution of the most relevant variables was normal. The ultrasound measurement protocol is very time-consuming and could be difficult to implement for routine assessment in clinical practice. However, ultrasound measurement is an effective and non-invasive method that provide very precise information with great interest. Regarding to the cognitive assessment, SPMSQ is an unspecific tool for assessing cognitive performance and could represent a limitation of the present study. In addition, the high variance in gender and not having split our data by sex can be considered a limitation of the present study. The lack of a control group of healthy active older adults could be a limitation. Future investigations with higher sample size and comparing echo intensity between groups of older adults with high and low scores in physical function evaluations could be interesting. 

## 5. Conclusions

We conclude that muscle thickness and echo intensity are related to strength in older adults; however, echo intensity is more associated with functional capacity than muscle thickness. Echo intensity can explain a significant percentage of the variability of functional capacity in older people. We found poor relationships between ultrasonography variables and cognitive tests. Functional capacity in institutionalized older adults could be explained by a deterioration of muscle quality assessed by echo intensity. Strategies to improve functional capacity in older adults should consider muscle echo intensity as an important variable due to its implications on functionality in older people. The main implication of the present study is that muscle echo intensity adjusted by handgrip strength could be used as an indirect and reversible indicator of functional capacity. Muscle ultrasound variables provide useful information for clinical practice in an integral assessment context in older adults.

## Figures and Tables

**Table 1:** Anthropometric, cognitive, physical functional, and ultrasonography characteristics.

Variable	Mean	SD	Variable	Mean	SD
Age (years)	85.4	7	GM MT (cm)	1.16	0.32
Height (m)	1.59	0.05	GM MVC MT (cm)	1.21	0.33
Weight (kg)	65.2	9.7	RF MT (cm)	1.26	0.27
BMI (kg/m^2^)	25.6	3.8	RF MVC MT (cm)	1.56	0.36
Mid-upper arm circumference (cm)	28.4	3.6	RL MT (cm)	1.21	0.21
Calf circumference (cm)	34.03	2.8	RL MVC MT (cm)	1.37	0.24
SPMSQ (0–12)	2.8	3.1	TA MT (cm)	2.3	0.29
Motor memory (0–10)	5.9	2.6	TA MVC MT (cm)	2.5	0.32
Tinetti (0–28)	19.2	6.6	WF MT (cm)	1.25	0.24
Barthel (0–100)	75.3	19.2	WF MVC MT (cm)	1.41	0.24
TUG (seconds)	20.8	11.5	BB EI (0–255)	82.46	12.65
GS (m/s)	0.6	0.1	GM EI (0–255)	69.36	11.86
SPPB (0–12)	5.3	3.1	RF EI (0–255)	69.62	8.51
HGS (kg)	16.5	4.7	RL EI (0–255)	79.68	11.66
Knee Extensors Strength (N)	135.1	62.9	TA EI (0–255)	87.93	7.47
BB MT (cm)	1.96	0.38	WF EI (0–255)	65.68	12.25
BB MVC MT (cm)	2.77	0.41			

BMI, body mass index; SPMSQ, Pfeiffer Short Portable Mental Status Questionnaire; TUG, timed-up-and-go; GS, gait speed; SPPB, Short Physical Performance Battery; STS, sit-to-stand; HGS, hand grip strength; BB, biceps brachii; MVC, maximum voluntary contraction; MG, medial gastrocnemius; RF, rectus femoris; RL, rectus lateralis, TA, tibialis anterior, WF, wrist flexors; EI, echo intensity; MT, muscle thickness.

**Table 2:** Pearson’s product–moment correlation coefficient between cognitive and physical variables and ultrasonography variables.

	UL EI	LL EI	UL MT	LL MT
SPPB	−0.323	−0.576 †	0.432	0.541 *
SPMSQ	−0.062	−0.274	0.030	0.007
Motor Memory	−0.280	−0.180	0.378	0.431
Barthel	−0.118	−0.307	0.344	0.582 ^†^
Tinetti	−0.052	−0.444 *	0.307	0.522 *
TUG (s)	0.211	0.344	−0.107	−0.266
GS (m/s)	−0.141	−0.556 *	0.219	0.327
HGS (kg)	−0.037	−0.268	0.562 ^†^	0.528 *
Knee Extensor Strength (N)	−0.523 *	−0.681 ^†^	0.515 *	0.495 *

* = *p* < 0.05, † = *p* < 0.01, SPPB, Short Physical Performance Battery; SPMSQ, Pfeiffer Short Portable Mental Status Questionnaire; TUG, Timed-Up-And-Go; GS, gait speed; HGS, hand grip strength; UL, upper limb; EI, echo intensity; LL, lower limb; MT, muscle thickness.

**Table 3:** Stepwise multiple regression analysis (*n* = 20).

Dependent Variable	Predictor Variables	Standardized β	Adjusted R^2^
SPPB			
Step 1	RF EI	−0.575 ^†^	0.293 ^†^
Step 2	RF EI	−0.481 *	0.449 ^†^
	HGS	0.431 *	
TUG			
Step 1	MG EI	0.567 *	0.265 *
			
Gait Speed			
Step 1	LLEI	−0.556 *	0.256 *
Step 2	LL EI	−0.426 *	0.585 ^†^
	HGS	0.594 ^†^	
Step 3	LLEI	−0.500 ^†^	0.731 ^†^
	HGS	0.532 ^†^	
	BMI	−0.390 *	
Motor memory			
Step 1	TA_MCV	0.634 ^†^	0.362 ^†^
			
SPMSQ			
Step 1	RF EI	−0.459 *	0.167 *

* = *p* < 0.05, † = *p* < 0.01 SPPB, Short Physical Performance Battery; RF, rectus femoris; EI, echo intensity; HGS, hand grip strength; TUG, timed-up-and-go; STS: sit-to-stand; LL: lower limb.

## Data Availability

The datasets used and/or analysed during the current study are available from the corresponding author on reasonable request.

## Abbreviations

Muscular thickness (MT); echo intensity (EI); Short Portable Mental Status Questionnaire (SPMSQ); Short Physical Performance Battery (SPPB); sit-to-stand (STS); timed-up-and-go test (TUG); handgrip strength (HGS); maximum voluntary contraction (MVC); wrist flexors (WF); biceps brachii (BB); rectus femoris (RF); vastus lateralis (VL); medial gastrocnemius (MG); and tibialis anterior (TA).
